# Healthcare utilization among informal caregivers of older adults in the Ashanti region of Ghana: a study based on the health belief model

**DOI:** 10.1186/s13690-023-01200-5

**Published:** 2023-10-23

**Authors:** Williams Agyemang-Duah, Mark W. Rosenberg

**Affiliations:** Department of Geography and Planning, Queen’s University, Kingston, ON K7L 3N6 Canada

**Keywords:** Health Belief Model, Healthcare utilization, Informal caregivers, Older adults, Ghana

## Abstract

**Background:**

Existing global evidence suggests that informal caregivers prioritize the health (care) of their care recipients (older adults) over their own health (care) resulting in sub-optimal health outcomes among this population group. However, data on what factors are associated with healthcare utilization among informal caregivers of older adults are not known in a sub-Saharan African context. Guided by the Health Belief Model (HBM), the principal objective of this study was to examine the association between the dimensions of the HBM and healthcare utilization among informal caregivers of older adults in the Ashanti Region of Ghana.

**Methods:**

Data were extracted from a large cross-sectional study of informal caregiving, health, and healthcare survey among caregivers of older adults aged 50 years or above (N = 1,853; mean age of caregivers = 39.15 years; and mean age of care recipients = 75.08 years) in the Ashanti Region of Ghana. Poisson regression models were used to estimate the association between the dimensions of the HBM and healthcare utilization among informal caregivers of older adults. Statistical significance of the test was set at a probability level of 0.05 or less.

**Results:**

The results showed that 72.9% (n = 1351) of the participants were females, 56.7% (n = 1051) were urban informal caregivers and 28.6% (n = 530)  had no formal education. The results further showed that 49.4% (n = 916) of the participants utilized healthcare for their health problems at least once in the past year before the survey. The final analysis showed a positive and statistically significant association between perceived susceptibility to a health problem (β = 0.054, IRR = 1.056, 95% CI = [1.041–1.071]), cues to action (β = 0.076, IRR = 1.079, 95% CI = [1.044–1.114]), self-efficacy (β = 0.042, IRR = 1.043, 95% CI = [1.013–1.074]) and healthcare utilization among informal caregivers of older adults. The study further revealed a negative and statistically significant association between perceived severity of a health problem and healthcare utilization (β= − 0.040, IRR = 0.961, 95% CI= [0.947-0.975]) among informal caregivers of older adults. The results again showed that non-enrollment in a health insurance scheme (β= − 0.174, IRR = 0.841, 95% CI= [0.774-0.913]) and being unemployed (β= − 0.088, IRR = 0.916, 95% CI= [0.850-0.986]) were statistically significantly associated with a lower log count of healthcare utilization among informal caregivers of older adults.

**Conclusion:**

The findings of this study to a large extent support the dimensions of the HBM in explaining healthcare utilization among informal caregivers of older adults in the Ashanti Region of Ghana. Although all the dimensions of the HBM were significantly associated with healthcare utilization in Model 1, perceived barriers to care-seeking and perceived benefits of care-seeking were no longer statistically significant after controlling for demographic, socio-economic and health-related variables in the final model. The findings further suggest that the dimensions of the HBM as well as demographic, socio-economic and health-related factors contribute to unequal healthcare utilization among informal caregivers of older adults in the Ashanti Region of Ghana.

**Table:** 

**Text box 1. Contributions to the literature**
• Data on factors influencing healthcare utilization among informal caregivers of older adults are unknown in Ghana which may delay the development of health policy and programmes targeting informal caregivers.
• This study highlights that the dimensions of the HBM, demographic, socio-economic and health-related variables contribute to unequal healthcare utilization among informal caregivers of older adults in the Ashanti Region of Ghana.
• This baseline study contributes to the empirical, methodological, theoretical and policy debates on healthcare utilization among informal caregivers of older adults in Ghana.
• More research on healthcare utilization among informal caregivers of older adults in sub-Saharan Africa (SSA) is necessary given the challenges governments are facing as population ages.

## Introduction

Informal caregivers provide care for the majority of older adults [1–8] especially given the rising number of older adults in national, regional, and international contexts and the associated health problems and healthcare system challenges [9–11]. Like care recipients, informal caregivers experience health problems [2, 12–14] that affect their health-related quality of life [15]. For instance, the available published works on the health of this sub-population suggest that informal caregivers have higher odds of suffering from depressive symptoms and poorer physical and mental health outcomes than those who do not provide care [13, 14, 16]. These dynamics provoke immediate discussion and research on health (care) seeking behaviour among informal caregivers.

Health (care) seeking behaviour is conceptualized as any action performed to address health problems and maintain good health [17–21]. Therefore, appropriate health (care) seeking behaviour can improve the overall health outcomes of informal caregivers [22]. That said, it is expected that informal caregivers would access and utilize frequent healthcare services to meet their growing healthcare needs considering their perceived poor health outcomes along with the health benefits of seeking appropriate healthcare [13, 14]. Unfortunately, most informal caregivers do not utilize healthcare services partly due to the demanding nature of their caregiving activities [23]. Evidence further indicates that informal caregivers put more priority on the health (care) of their care recipients at the expense of their own health (care) [13, 24–26]. Besides, most caregivers have limited access to and use less healthcare due to lack of financial and social resources to cover the often-catastrophic healthcare cost [27]. Moreover, one study has highlighted that most healthcare delivery models largely tend to concentrate on care recipients and do not provide support for informal caregivers [28]. In this context, studies have consistently reported that informal caregivers have higher unmet health (care) needs [29–33].

It is important to acknowledge that few studies have focused on healthcare utilization among informal caregivers in developed countries [10, 26, 34]. Yet these studies are limited in terms of geographical, health conditions, theoretical and conceptual scope [10, 34–36]. For instance, in a national survey on healthcare utilization between informal caregivers and non-caregivers in the United States, Shaffer and Nightingale [26] found that there was no statistically significant difference between caregivers and non-caregivers regarding the number of healthcare appointments in the past year. In their study on psychiatric disorders and mental health services use among caregivers of advanced cancer patients, Vanderwerker et al. [10] highlighted that 46% of caregivers with a current psychiatric disorder access mental health services in the United States. The study further revealed that caregivers who discuss their mental health concerns before and after were more likely to access mental health interventions [10]. In another US-based study on understanding patterns of service utilization among informal caregivers of community older adults, Hong et al. [35] established that on average, caregivers of older adults utilized 1.7 services with assistive device service, personal or nursing care and house modifications being the prevalent services used. Their study further indicated that caregiver’s network compositions predict service use patterns among informal caregivers of older adults. Further, in their longitudinal study on healthcare use and cost in dementia caregivers in the United States, Zhu et al. [36] highlighted that patients’ increased comorbidity conditions and dependence explained increased healthcare utilization by their caregivers. Beyond that, a rise in caregiver depressive symptoms was associated with an increase in healthcare use and costs among family caregivers [36]. In a systematic study, Bieber et al. [34] reported that factors such as ethnicity, gender, place of residence and attitude towards care services are associated with access to and use of formal community care by people with dementia and their caregivers in North America, Europe, Australia, and Asia.

However, these existing empirical and systematic studies on healthcare utilization among informal caregivers employed a small sample size [26], were limited to caregivers of advanced cancer patients [10], informal caregivers with dementia [36], caregivers who utilize mental health services [10], informal caregivers in general [26], informal caregivers of older adults in the United States [35] as well as North America, Europe, Australia, and Asia [34]. They were also atheoretical [10, 26, 34, 36]. Although, these studies provide important information on healthcare utilization among informal caregivers in developed countries, the generalization of findings from these studies to SSA context is not possible because of differences in socio-cultural factors and healthcare systems.

There are no known published data on the prevalence of healthcare utilization and associated factors among informal caregivers of older adults in SSA, particularly in Ghana. It is important to mention that studies on healthcare utilization that exist in Ghana have focused on the general population [37–40], pregnant women [41–43], women in general [44], as well as older adults [45–50] but not informal caregivers of older adults. What is known from these Ghanaian studies is that demographic, socio-economic [37, 45, 47, 51], health-related [47], system (such as waiting time at the healthcare facility and healthcare costs), client preference (such as sources of funds for healthcare and sources of healthcare information) and institutional (including communication problems and level of healthcare treatment) [52] factors explain healthcare utilization. Thus far, the associations between the dimensions of the HBM and healthcare utilization among informal caregivers of older adults in Ghana are unknown. Taking into consideration the crucial role that caregivers perform in the delivery of care to older adults, it is necessary to understand their healthcare utilization to promote their wellbeing [10]. A baseline understanding of this knowledge area is also important to guide health policy development and programmes to improve healthcare utilization among informal caregivers of older adults in Ghana. Using data from a large cross-sectional survey on informal caregiving, health and healthcare among caregivers of older adults in the Ashanti Region of Ghana, the objectives of this study are as follows: (1) to determine the dimensions of the HBM associated with healthcare utilization among informal caregivers of older adults (2) to determine if the dimensions of the HBM still determine healthcare utilization among informal caregivers of older adults after controlling for demographic, socio-economic and health-related factors.

## Health belief model (HBM)

In this study, the HBM provides a theoretical framework to investigate healthcare utilization among informal caregivers of older adults. Since its development, the HBM continues to constitute a key theoretical framework for research focusing on health behaviour [53–58] and has predicted a range of behaviours [58]. Developed in the early 1950s by a social psychologist at the United States’ Public Health Service, the HBM seeks to understand why people fail or accept to seek early interventions for diseases [53, 59–61]. Beyond that, the HBM has subsequently been employed to explain individuals’ response to symptoms and medical compliance [62].

The central dimensions of the HBM are perceived susceptibility, perceived severity, perceived benefits, perceived barriers, cues to action (health motivation) [53, 60, 61, 63–65] and self-efficacy [60, 64–66]. The HBM conceptualizes that individuals are more likely to engage in a particular health behaviour if they: (1) perceive that they are vulnerable to a health problem-perceived susceptibility to a health problem; (2) believe the health problems have negative implications on their activities of daily living- perceived severity of a health problem; (3) believe that the intervention (such as care-seeking) will be effective in reducing the health problems- perceived benefits of care-seeking; (4) perceive that there are few barriers to performing an action to address the health problem- perceived barriers to care-seeking [60, 67–69]; (5) perceive that some internal (such as symptoms) and external (such as mass media, healthcare providers, family members and friends ) factors influence the decision-making process of care-seeking - cues to action [53, 60, 68]; (6) perceive that they can perform or seek an action (such as care-seeking) through their own- self-efficacy [70]. In this context, these health belief dimensions are likely to determine healthcare utilization among informal caregivers of older adults.

Based on the theorization of the HBM, the following hypotheses are examined in this study: (1) perceived susceptibility to a health problem is positively and significantly associated with healthcare utilization; (2) perceived severity of a health problem is positively and significantly associated with healthcare utilization; (3) there are positive and significant associations between perceived benefits of care-seeking and healthcare utilization; (4) there are negative and significant associations between perceived barriers to care-seeking and healthcare utilization; (5) the association between cues to action and healthcare utilization achieves positive and statistical significance; (6) self-efficacy is positively and significantly associated with healthcare utilization.

Janz and Becker [53] argue that apart from the above dimensions of the HBM, demographic, socio-psychological and structural factors may influence health-related behaviour. For instance, evidence suggests that demographic, socio-economic [18, 34, 71] and health-related factors [18] are associated with healthcare utilization. Considering this, one critical question is, will all the dimensions of the HBM still predict healthcare utilization among informal caregivers of older adults after controlling for demographic, socio-economic and health-related factors?

It is important to mention that the existing published works that applied the HBM were conducted in different geographical regions and population groups, and none focused on informal caregivers of older adults. For instance, Leavitt [72] employed the HBM to determine the utilization of ambulatory care services and reported that the single best predictor of utilization is a person’s belief of their susceptibility to diseases, followed by their belief of benefits associated with preventive health. Luquis and Kensinger [64] applied the HBM to assess preventive services use among young adults in the United States. Their study revealed that perceived susceptibility and perceived seriousness play a role in the utilization of health preventive services among young adults. In Tanzania, Tungaraza and Joho [73] employed the HBM and self-determination theory to explain the use of antenatal care services. They reported that low perceived barriers are associated with antenatal care visits in Tanzania. Even though, these published works focused on different population groups and geographical regions, they [the works] provide a baseline foundation to employ the HBM as a theoretical framework to examine healthcare utilization among informal caregivers of older adults in Ghana. Also, using the HBM may offer a theoretical perspective for the study and position our results within this theoretical context [47].

## Methods

### Study design

**Fig. 1:**
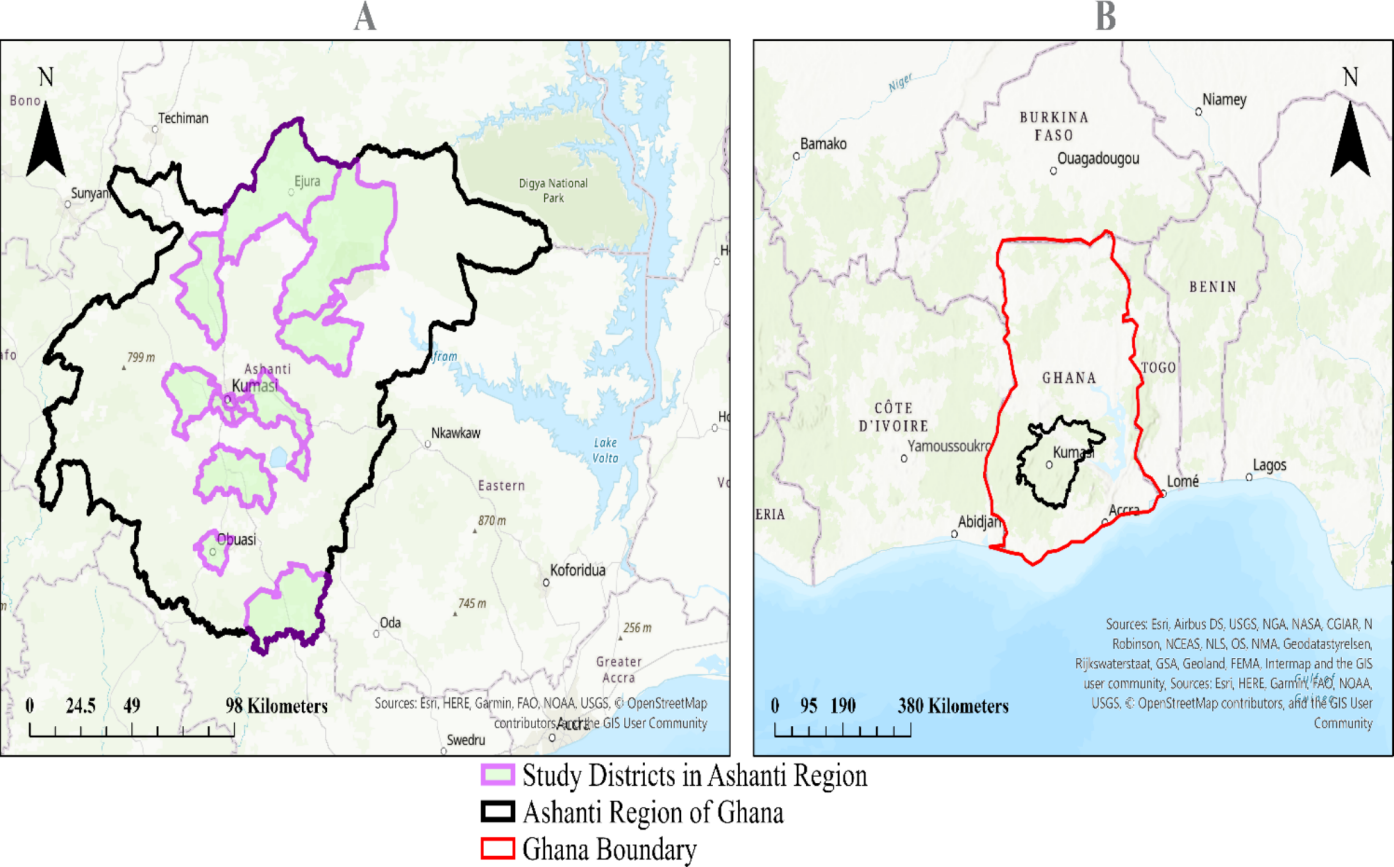
Study area location. (**A**) shows the study area covered by the selected districts, and (**B**) shows the study area in the context of Ghana and neighboring countries in SSA

This study used data from a large cross-sectional survey on informal caregiving, health, and healthcare among caregivers of older adults aged 50 years or more residing in 13 districts in the Ashanti Region of Ghana (see Fig. 1). The study which focused on informal caregivers of older adults was a one-time survey (that is, data collection took place at one time point between July and September 2022) [74, 75].

### Sample size and sampling procedure

The study employed both probability (such as cluster and simple random) and non-probability (such as snowball) sampling techniques to select the study sites and the participants. It is important to highlight that the Ashanti Region has 43 districts (including Metropolitan and Municipals) of which 13 of them were randomly included in this study. A cluster sampling technique was employed to divide the study region into three geographical zones (northern, middle, and southern zones) by taking into consideration geographical location, socio-economic status, and cultural differences. Subsequently, to give all the districts an equal chance of being selected, a simple random sampling technique was used to choose specific numbers of districts from each of the demarcated zones. More specifically, we randomly chose 3 districts each from the northern (Offinso Municipal, Ejura-Sekyedumase Municipal and Sekyere Central District) and southern zones (Adansi-South District, Bekwai Municipal and Obuasi Municipal) and 7 districts from the middle zone (Kumasi Metropolis, Atwima Nwabiagya Municipal, Sekyere-Kumawu District, Ejisu Municipal, Kwadaso Municipal, Asokwa Municipal and Oforikrom Municipal). This is because the middle zone has a greater number of districts compared to the northern and southern zones. The approach was to ensure that the data were robust and representative of the views of informal caregivers on healthcare utilization in the study area.

More specifically, the procedures for the selection of the districts are as follows. First, the names of the districts in each of the demarcated zones were written on a piece of paper. Second, they [the districts] were put in three different boxes (one for each of the demarcated zones). Third, we determined the number of districts that were to be selected from each of the three boxes. Last, a blind folded person chose the required number of districts allotted for each demarcated zone until the required number of the districts for each of the zone was obtained.

Then 3 communities were chosen from each of the 13 districts making 39 communities. Out of the 13 districts, 4 of them (Kumasi Metropolis, Asokwa Municipal, Oforikrom Municipal and Kwadaso Municipal) have purely urban communities. All 3 communities that were randomly selected in these 4 districts were urban (3 urban communities by 4 districts making 12 urban communities). The remaining 9 districts have both urban and rural communities. Given this, we randomly selected 1 urban community and 2 rural communities from these remaining districts (1 urban community by 9 districts making 9 urban communities; 2 rural communities by 9 districts making 18 rural communities). In this study, more urban communities were included because of diversities of the demographics, socio-economic and cultural characteristics of the urban informal caregivers in the sampled study districts. Hence, to account for these differences, there was a need to include more urban communities than rural communities in this study. An additional rationale was to ensure that the findings were representative and could be generalized to reflect the view of both rural and urban informal caregivers of older adults in relation to healthcare utilization in the study area. In all, 21 urban (9 + 12) and 18 rural communities were included in this study.

An estimated number of 1,900 informal caregivers were recruited to participate in the study. A formula, n = design effect × [(Zα/2)^2^ ×P (1-P)]/ε^2^ by Lwanga and Lemeshow (1991) with a design effect of 1.5, a confidence interval of 95% (Zα/2 = 1.96), and margin of error of 4% were used to calculate the minimum sample size. Considering that the actual number of informal caregivers of older adults is unknown in Ghana/and or the study area, we used the conservative prevalence (p) of 0.48. Inserting the above parameters in the formula, a minimum sample size of 899 participants was obtained. However, after arriving at the minimum sample size, we oversampled the participants to get a final sample size of 1,900 participants. The decision was to minimize the potential effect the use of snowball sampling could have on the statistical rigor and representativeness of our study. Of those sampled, 36 (1.89%) of the participants refused to participate in the study, 7 (0.37%) of them offered unfinished responses and 4 (0.21%) of the participants’ responses included missing data, yielding a response rate of 97.52%. Thus, the analytical sample for this study was restricted to 1853 participants. We emphasize that the missing data and the unfinished responses were excluded from the final analysis.

We used snowball sampling to recruit the participants because we did not have records of the number of informal caregivers of older adults in Ghana and the study area. The process of recruiting the participants began by contacting community leaders and discussing the purpose of the study with them. Because they hailed from the study communities, they were able to identify the participants. Also, current participants recommended potential participants who provide informal care for older adults to the research team to take part in the survey.

### Inclusion and exclusion criteria

This study employed both inclusion and exclusion criteria in recruiting the study participants. Key among the inclusion criteria was that a participant should be: (1) an informal caregiver who provides care for older adults who are 50 years or above; (2) a family member, non-family member and/or friend providing care for older adults; (3) 18 years or more; (4) providing informal care for at least one year; (5) providing care for an older person at least a day in a week; 5) living in rural areas and providing care for an older family member, friend or neighbour in villages or rural communities; (6) residing in urban areas and providing care for an older family member, friend or neighbour in cities or urban communities. By providing informal care for at least one year, an individual could share their views on their health beliefs and healthcare utilization. The only exclusion criterion was that informal caregivers of older persons aged 50 years or above who were sick during the period of data collection were not included in the study. This is because, those people were considered not fit enough to provide quality data for the study.

### Data collection procedure

An interviewer-administered questionnaire was used to collect data. Questions that were covered in the survey included demographic, socio-economic, health-related, dimensions of the HBM, and healthcare utilization dynamics, among others. All the questions in the questionnaire were later entered into Qualtrics e-survey tool which helped us to digitally record the responses of the participants. The questionnaire was developed in English but was read in Twi (the local language of the participants). The questionnaire was administered to participants who could not read and/or write by trained research assistants at their preferred locations and in the participant’s preferred language. To ensure data quality, the research assistants were graduate students from the Kwame Nkrumah University of Science and Technology (KNUST), Ghana with diverse knowledge in Medical and Health Geography as well as Public Health. They have also served as field researchers with over 3 years experience of collecting data for studies with large sample sizes. They were further trained in research ethics and signed confidentiality agreements before the field work began. They were closely supervised and constantly reminded of the ethical protocols guiding the study. The data collection took place in the various homes of the participants and lasted between 30 and 35 min. We attest that the process of the data collection was free from the interference of any third party. Approved by the institutional review board of the authors, participants were compensated with a cake of soap, and they had the opportunity to withdraw at any point before or during the administration of the questionnaire. They could also refuse to respond to any of the questions without affecting their compensation.

### Ethical clearance and consent

Ethical clearance was sought from appropriate institutions and committees in accordance with the Helsinki declaration. First, the Ashanti Regional Health Directorate under the Ghana Health Service provided approval to the study region for field work to begin (Ref: GHS/ASH/RES/V.2). Further, ethical approval was sought from the General Research Ethics Board at Queen’s University (GREB**)**, Kingston, Canada (Ref: GGEOPL-344-22**)** and the Committee on Human Research Publication and Ethics (CHRPE), School of Medical Sciences, College of Health Sciences, KNUST, Kumasi, Ghana **(**Ref: CHRPE/AP/182/22). Informed consent consisting of both oral and written consent was sought from the participants. The procedure for obtaining verbal informed consent was approved by the ethics committee/institutional review boards above. We further confirm that for illiterate participants, informed consent to participate in the study was obtained from their legal guardians.

### Dependent variable

In this study, our outcome variable was healthcare utilization. Healthcare utilization was defined as seeking treatment from a formal healthcare provider (including public/private hospitals, clinics, and health centres) [18, 45–47, 51, 76], and an informal healthcare provider (such as over-the-counter medication, salespeople, drug peddlers, traditional treatment/unlicensed practitioners, paraprofessional, and self-care treatment) [51] over the last one year preceding the survey. The one-year estimation rate of healthcare utilization in this study is consistent with earlier published works [45–47, 51, 77, 78].

Considering that previous studies have limited their conceptualization of healthcare utilization to only formal healthcare  [18, 46, 76], measuring healthcare utilization in this study based on two key dimensions of healthcare (formal healthcare and informal healthcare providers) offers us the opportunity to provide a comprehensive measurement of healthcare utilization among informal caregivers to inform broader health policy development and programmes. Specifically, we asked the question; how many times have you sought treatment for your health problem in the last year? The responses were operationalized as 0 = None,  1 = Once,  2 = 2 times, 3 = 3 times, 4 = 4 times, 5 = 5 or more times. Unlike some previously published works in Ghana and elsewhere which measured healthcare utilization as a dichotomous variable [18, 47, 51, 76], following Asante et al. [45], Gyasi et al. [77] as well as Shaffer and Nightingale [26], we measured healthcare utilization as a count variable, that is, the number of times that a participant has sought treatment for a health problem. We argue that unlike a dichotomous variable, a count variable is more likely to minimize the possibility of losing important information in the analysis while at the same time increasing the statistical rigor of the association between the dimensions of the HBM and healthcare utilization.

### Independent and control variables

Based on the HBM, our primary predictor variables were perceived susceptibility to a health problem, perceived severity of a health problem, perceived benefits of care-seeking, perceived barriers to care-seeking, cues to action and self-efficacy and were all measured as continuous variables. Following the scales of Shmueli [79], Zhao et al. [80] Champion [81], Champion [82], Maiman et al. [83] and Rosenstock [63], we developed a 16-item scale to measure the various dimensions of the HBM. First, we measured perceived susceptibility to a health problem using a 2-item scale based on the following: (a) “*It is extremely likely that I will sustain a health problem”* (b) *“I am more likely than other informal caregivers to get a health problem”.* Consistent with Moorthy et al. [84], Shiryazdi et al. [85] and Champion [81], the responses were on a 5-point response scale (1 = Disagree strongly, 2 = Disagree a little, 3 = Neither agree nor disagree, 4 = Agree a little, 5 = Agree strongly) and the same response scale was used for the other dimensions of the HBM. The composite score ranged from 2 to 10 with a higher score indicating higher perceived susceptibility to a health problem and vice versa. Second, we employed a 3-item scale to assess the perceived severity of a health problem taking into consideration the following criteria: (a) *“The thought of a health problem scares me”*; (b) “*Having a health problem would threaten the relationship with my family*”; (c) *“If I had a health problem, my whole life would change”*. The composite score ranged from 3 to 15 with a higher score indicating higher perceived severity of a health problem and vice versa. Third, we determined the perceived benefits of care-seeking based on a 3-item scale. These are: (a) “*When I access healthcare, I feel good about myself”*; (b) “*Use of healthcare would improve my health-related quality of life”;* (c) *“If I access healthcare, it will decrease my chances of getting a health problem”*. The composite score ranged from 3 to 15 with a higher score indicating higher perceived benefits of care-seeking and vice versa. Fourth, we used a 2-item scale to assess perceived barriers to care-seeking based on these criteria: (a) *“Accessing healthcare would take too much time”*; (b) *“I don’t have the resources (funds/support) to access healthcare*”. The composite score ranged from 2 to 10 with a higher score demonstrating higher perceived barriers to care-seeking and vice versa. Fifth, we conceptualized cues to action based on a 3-item scale: (a) “*I want to discover health problems early”*; (b) “*Maintaining good health is extremely important to me*”; (c) “*I feel it is important to carry out activities which will improve my health”*. The composite score ranged from 3 to 15 with a higher score implying higher cues to action and vice versa. Last, we determined self-efficacy using the following 3-item scale: (a) *“I am able to tell I have a health problem”* (b) “*I am able to tell where to seek for healthcare”* (c) *“I am able to tell when I need healthcare”.* The aggregate score ranged from 3 to 15 with a higher score showing higher self-efficacy and vice versa. We calculated a Cronbach’s Alpha value of 0.894 suggesting strong internal consistency for the overall 16-item scale covering all the dimensions of the HBM as highlighted in Table 1. As a result, we argue that the scale used to measure the dimensions of the HBM is culturally and contextually relevant in the study area.

**Table 1:** Reliability statistics on all the dimensions of the HBM

Dimensions of the HBM	Cronbach’s Alpha Based on Standardized items	Number of items
Perceived susceptibility to a health problem	0.847	2
Perceived severity of a health problem	0.864	3
Perceived benefits of care-seeking	0.891	3
Perceived barriers to care-seeking	0.588	2
Cue to action	0.811	3
Self-efficacy	0.860	3
All the dimensions of the HBM	0.894	16

We also controlled for demographic, socio-economic and health-related variables. This is because, evidence suggests that apart from the dimensions of the HBM, demographic, socio-economic and health-related factors explain health-related behaviour (including healthcare utilization) [53]. Multicollinearity analysis on the independent and control variables is reported. We had a value of less than 2.2 for each of the independent and control variables indicating no strong multicollinearity as shown in Table 2. Demographic variables included place of residence (0 = rural, 1 = urban), age (years) (0 = 18–24; 1 = 25–34; 2 = 35–44; 3 = 45–54; 4 = 55–64; 5 = 65 or above), gender (0 = male; 1 = female) and marital status of caregivers (0 = never married, 1 = currently married, 2 = separated/widowed/divorced). Socio-economic variables included employment status (0 = unemployed, 1 = employed), income level (GH¢) (0 = less than 1000 [US$99.50 as of the time of the field survey, September 2022], 1 = 1000–1999, 2 = 2000 or above), education level (0 = no formal education, 1 = primary education, 2 = junior high school education, 3 = senior high school education, 4 = tertiary education) and health insurance enrollment of caregivers (0 = no; 1 = yes). A health-related variable included self-rated health of caregivers (0 = very poor/poor, 1 = fair, 2 = good, 3 = very good, 4 = excellent). The inclusion of these specific control variables was informed by the existing literature on healthcare utilization and its associated factors [37–40, 45–51]. Place of residence, gender, employment status and health insurance enrollment of caregivers were measured as dichotomous variables. Income, age, education, and self-rated health of caregivers were measured as ordinal variables. The marital status of caregivers was measured as a nominal variable.

**Table 2:** Multicollinearity Statistics

Variables	Tolerance	Variance Inflation Factor (VIF)
Place of residence of caregivers	0.934	1.071
Age (years) of caregivers	0.629	1.590
Gender of caregivers	0.894	1.118
Marital status of caregivers	0.677	1.478
Employment status of caregivers	0.888	1.126
Income (GH¢) of caregivers	0.940	1.064
Education level of caregivers	0.763	1.311
Health insurance enrollment of caregivers	0.871	1.148
Self-rated health of caregivers	0.900	1.111
Perceived susceptibility to a health problem	0.628	1.594
Perceived severity of a health problem	0.551	1.814
Perceived benefits of care-seeking	0.472	2.120
Perceived barriers to care-seeking	0.656	1.525
Cues to action	0.564	1.772
Self-efficacy	0.600	1.668
Mean (Average) value of Tolerance/VIF	0.73	1.434
Minimum value of Tolerance/VIF	0.472	1.064
Maximum value of Tolerance/VIF	0.940	2.120

### Analytical strategy

All the analyses used the SPSS version 28 (IBM Armonk, NY) software. Descriptive statistics including means, standard deviations, percentages, and frequencies were used to describe (1) the background characteristics of the participants (2) the dimensions of the HBM (3) the dynamics of healthcare utilization. Because the dependent variable was count data, we employed Poisson regression models as our inferential analytical framework. We specifically developed four different models. Model 1 included all the dimensions of the HBM. In Model 2, we added demographic variables to all variables in Model (1). In Model 3, we added socio-economic variables to all variables in Model (2). In Model 4 (the final model), we included a health-related variable to all variables in Model (3). We reported beta values, incidence rate ratio (IRR) and confidence intervals (CI) for each variable category associated with healthcare utilization. The significance level of the test was set at a probability value of 0.05 or less.

## Results

### Sample characteristics of the participants

The sample characteristics of the participants are reported in Table 3. We re-emphasize that the analytical sample size for this study is 1,853 participants. With the exception of some of the variables in Table 4, all other variables have a sample size of 1,853 participants. The results showed that 27.7% (n = 513) of the participants were aged between 25 and 34 years, 72.9% (n = 1,351) were females, 56.7% (n = 1,051) were in urban areas, 28.6% (n = 530) had no level of formal education, 66.4% (n = 1,231) were employed, 55.8% (n = 1,034) were married, 76.8% (n = 1,422) earned less than GH¢1,000 in a month, 76.6% (n = 1,419) were enrolled in a health insurance scheme and 47.9% (n = 887) self-rated their health as very good.

**Table 3:** Sample characteristics of the participants (N = 1,853)

Variables	Category	Count	%
Age (years) of caregivers	18–24	266	14.3
	25–34	513	27.7
	35–44	439	23.7
	45–54	369	19.9
	55–64	172	9.3
	65 or above	94	5.1
Gender of caregivers	Male	502	27.1
	Female	1351	72.9
Residence of caregivers	Rural	802	43.3
	Urban	1051	56.7
Education level of caregivers	No formal education	530	28.6
	Primary	152	8.2
	Junior High School	445	24.0
	Senior high school	445	24.0
	Tertiary	281	15.2
Employment status of caregivers	Unemployed	622	33.6
	Employed	1231	66.4
Marital status of caregivers	Never married	561	30.3
	Currently Married	1034	55.8
	Separated/Widowed/ Divorced	258	13.9
Monthly income (GH¢) of caregivers	Less than 1000	1422	76.8
	1000–1999	299	16.1
	2000 or above	132	7.1
Health insurance enrollment of caregivers	No	434	23.4
	Yes	1419	76.6
	Very poor/poor	28	1.5
Self-rated health of caregivers	Fair	72	3.9
	Good	353	19.0
	Very good	887	47.9
	Excellent	513	27.7

**Table 4:** Descriptive statistics on items on the dimensions of the HBM  (N = 1,853)

Dimensions/items	Response	Count	%	Mean	SD
**Perceived susceptibility to a health problem**					
It is extremely likely I will sustain a health problem	Disagree Strongly	286	15.5	3.71	1.573
	Disagree a little	275	14.8		
	Neither agree nor disagree	89	4.8		
	Agree a little	235	12.7		
	Agree strongly	968	52.2		
I am more likely than other informal caregivers to get a health problem	Disagree Strongly	379	20.5	3.46	1.642
	Disagree a little	291	15.7		
	Neither agree nor disagree	124	6.7		
	Agree a little	219	11.8		
	Agree strongly	840	45.3		
**Perceived severity of a health problem**					
The thought of a health problem scares me	Disagree Strongly	102	5.5	4.35	1.082
	Disagree a little	72	3.9		
	Neither agree nor disagree	44	2.4		
	Agree a little	486	26.2		
	Agree strongly	1149	62.0		
Having a health problem would threaten the relationship with my family/care recipient	Disagree Strongly	105	5.7	4.39	1.070
	Disagree a little	54	2.9		
	Neither agree nor disagree	47	2.5		
	Agree a little	447	24.1		
	Agree strongly	1200	64.8		
If I had a health problem, my whole life would change	Disagree Strongly	70	3.8	4.50	0.957
	Disagree a little	53	2.9		
	Neither agree nor disagree	37	2.0		
	Agree a little	420	22.6		
	Agree strongly	1273	68.7		
**Perceived benefits of care-seeking**					
When I access healthcare, I feel good about myself	Disagree Strongly	45	2.4	4.58	0.802
	Disagree a little	22	1.2		
	Neither agree nor disagree	34	1.9		
	Agree a little	460	24.8		
	Agree strongly	1292	69.7		
Use of healthcare would improve my health-related quality of life	Disagree Strongly	39	2.1	4.63	0.744
	Disagree a little	15	0.8		
	Neither agree nor disagree	18	1.0		
	Agree a little	445	24.0		
	Agree strongly	1336	72.1		
	Disagree Strongly	47	2.5	4.63	0.792
If I access healthcare, it will decrease my chances of getting a health problem	Disagree a little	20	1.1		
	Neither agree nor disagree	22	1.2		
	Agree a little	398	21.5		
	Agree strongly	1366	73.7		
**Perceived barriers to care-seeking**					
Accessing healthcare would take too much time	Disagree Strongly	214	11.5	4.04	1.380
	Disagree a little	128	6.9		
	Neither agree nor disagree	64	3.5		
	Agree a little	418	22.6		
	Agree strongly	1029	55.5		
I don’t have the resources (funds/support) to access healthcare	Disagree Strongly	57	3.1	4.42	0.981
	Disagree a little	83	4.5		
	Neither agree nor disagree	75	4.0		
	Agree a little	447	24.1		
	Agree strongly	1191	64.3		
**Cues to action**					
I want to discover health problems early	Disagree Strongly	25	1.3	4.66	0.651
	Disagree a little	7	0.4		
	Neither agree nor disagree	14	0.8		
	Agree a little	476	25.7		
	Agree strongly	1331	71.8		
Maintaining good health is extremely important to me	Disagree Strongly	9	0.5	4.75	0.519
	Disagree a little	3	0.1		
	Neither agree nor disagree	14	0.8		
	Agree a little	384	20.7		
	Agree strongly	1443	77.9		
I feel it is important to carry out activities which will improve my health	Disagree Strongly	10	0.5	4.77	0.512
	Disagree a little	2	0.1		
	Neither agree nor disagree	11	0.6		
	Agree a little	365	19.7		
	Agree strongly	1465	79.1		
**Self-efficacy**					
I am able to tell I have a health problem	Disagree Strongly	6	0.3	4.65	0.583
	Disagree a little	12	0.7		
	Neither agree nor disagree	33	1.8		
	Agree a little	519	28.0		
	Agree strongly	1283	69.2		
I am able to tell where to seek for healthcare	Disagree Strongly	9	0.5	4.66	0.575
	Disagree a little	8	0.4		
	Neither agree nor disagree	22	1.2		
	Agree a little	517	27.9		
	Agree strongly	1297	70.0		
I am able to tell when I need healthcare	Disagree Strongly	7	0.4	4.69	0.542
	Disagree a little	4	0.2		
	Neither agree nor disagree	19	1.0		
	Agree a little	503	27.2		
	Agree strongly	1320	71.2		

### Descriptive analysis of the dimensions of the health belief model

The descriptive analysis of the dimensions of the HBM is reported in Table 5. On the perceived susceptibility to a health problem, the majority (52.2%, n = 968) of the participants ‘agreed strongly’ that it was extremely likely that they would sustain a health problem. Also, 45.3% (n = 840) of the participants ‘agreed strongly’ that they were more likely than other informal caregivers to get a health problem. Regarding the perceived severity of a health problem, 62% (n = 1,149) of the participants ‘agreed strongly’ that the thought of a health problem scared them, 64.8% (n = 1,200) ‘agreed strongly’ that having a health problem would threaten their relationships with the family/care recipient and 68.7% (n = 1,273) ‘agreed strongly’ that if they had a health problem, their whole life would change. Concerning the perceived benefits of care-seeking, 69.7% (n = 1,292) ‘agreed strongly’ that when they accessed healthcare, they felt good about themselves, 72.1% (n = 1,336) ‘strongly agreed’ that the use of healthcare would improve their health-related quality of life and 73.7% (n = 1,366) ‘agreed strongly’ that if they accessed healthcare, it would decrease their chances of getting a health problem. On the perceived barriers to care-seeking, 55.5% (n = 1,029) of the participants ‘agreed strongly’ that accessing healthcare would take too much time and 64.3% (n = 1,191) ‘agreed strongly’ that they did not have the resources (funds/support) to access healthcare. Regarding cues to action, 71.8% (n = 1,331) of the participants ‘agreed strongly’ that they wanted to discover health problems early, 77.9% (n = 1,443) ‘agreed strongly’ that maintaining good health was extremely important to them and 77.9% (n = 1,465) felt that it was important to carry out activities which would improve their health. Regarding self-efficacy, 69.2% (n = 1,283) of the participants ‘agreed strongly’ that they were able to tell they have a health problem, 70% (n = 1,297) ‘agreed strongly’ that they were able to tell where to seek healthcare and 71.2% (n = 1,320) ‘agreed strongly’ that they were able to tell when they need healthcare.

**Table 5:** Dynamics of healthcare utilization (N = 1,853)

Variable (s)	Response	Count	%
How many times have you sought healthcare for your health problem in the last 1 year?	None	937	50.6
	Once	249	13.5
	2 times	326	17.6
	3 times	143	7.7
	4 times	60	3.2
	5 or more times	138	7.4
	**Total**	**1853**	**100**
From where did you seek healthcare in the last 1 year? Self-care/self-treatment	No	625	68.2
	Yes	291	31.8
	**Total**	**916**	**100**
From where did you seek healthcare in the last 1 year? Drugstore/chemical shop Salespeople	No	450	49.1
	Yes	466	50.9
	**Total**	**916**	**100**
From where did you seek healthcare in the last 1 year? Traditional treatment	No	699	76.3
	Yes	217	23.7
	Total	916	100
From where did you seek healthcare in the last 1 year? Paraprofessional	No	908	99.1
	Yes	8	0.9
	**Total**	**916**	**100**
From where did you seek healthcare in the last 1 year? Allopathic provider/facility	No	338	36.9
	Yes	578	63.1
	Total	916	100
What type of health provider or facility did you consult most?	Public health facility	698	76.2
	Private health facility	218	23.8
	**Total**	**916**	**100**
How early did you seek healthcare for health problems after detecting the symptom?	Immediately/day	319	34.8
	2 days	227	24.8
	3 days	174	19.0
	4 days	69	7.5
	5 days	31	3.4
	6 days	8	0.9
	One week or more	88	9.6
	**Total**	**916**	**100**

### Healthcare utilization dynamics among informal caregivers of older adults

The healthcare utilization among informal caregivers of older adults has been reported in Table 4. The results showed that 49.4% (n = 916) had sought healthcare for their health problems at least once in the past year before the survey. In a multiple-response question which sought to find out from the participants about where they sought healthcare, the results showed that 63.1% (n = 578) sought healthcare from a healthcare facility, 50.9% (n = 466) sought healthcare from a drugstore/chemical shop, 31.8% (n = 291) sought self-treatment, 23.7% (n = 217) sought traditional treatment and 0.9% (n = 8) sought healthcare from paraprofessionals. The study further revealed that 76.2% (n = 698) of the participants sought healthcare from a public health facility whereas 23.8% (n = 218) sought treatment from a private health provider. When asked how early the participants sought healthcare for health problems, 34.8% (n = 319) responded they sought healthcare immediately/day after detecting the symptoms.

### Factors associated with healthcare utilization among informal caregivers of older adults

In Model 1, the results showed that perceived susceptibility to a health problem was positively and significantly associated with healthcare utilization (β = 0.067, IRR = 1.069, 95% CI= [1.054–1.083]). Also, the perceived severity of a health problem was negatively and significantly associated with healthcare utilization (β= − 0.038, IRR = 0.963, 95% CI= [0.949-0.979]). Moreover, perceived benefits of care-seeking were positively and significantly associated with healthcare utilization (β = 0.023, IRR = 1.023, 95% CI= [1.002–1.045]). There was a negative and significant association between perceived barriers to care-seeking and healthcare utilization (β= − 0.021, IRR = 0.980, 95% CI= [0.961-0.998]). In addition, there was a positive and significant association between cues to action and healthcare utilization (β = 0.064, IRR = 1.066, 95% CI= [1.032-1.100]). Self-efficacy was positively and significantly associated with healthcare utilization (β = 0.035, IRR = 1.035, 95% CI= [1.006–1.066]).

In Model 2, after adding demographic variables to all variables in Model 1, we observed that apart from perceived benefits of care-seeking, all the other dimensions of the HBM achieved statistical significance in relation to healthcare utilization. Specifically, we reported a positive and statistically significant association between perceived susceptibility to a health problem and healthcare utilization (β = 0.059, IRR = 1.061, 95% CI= [1.047–1.076]). There was also a negative and statistically significant association between perceived severity of a health problem and healthcare utilization (β= − 0.036, IRR = 0.965, 95% CI= [0.951–0.978]). A negative and statistically significant association between perceived barriers to care-seeking and healthcare utilization was reported (β= − 0.025, IRR = 0.976, 95% CI= [0.957–0.994]). There was a positive and statistically significant association between cues to action and healthcare utilization (β = 0.064, IRR = 1.066, 95% CI= [1.033–1.101]). Self-efficacy was positively and significantly associated with healthcare utilization (β = 0.033, IRR = 1.033, 95% CI= [1.004–1.064]).

In Model 3, when we included socio-economic factors with all variables in Model 2, except for perceived benefits of care-seeking, all the other dimensions of the HBM were significantly associated with healthcare utilization. For instance, there was a positive and statistically significant association between perceived susceptibility to a health problem and healthcare utilization (β = 0.051, IRR = 1. 053, 95% CI= [1.038–1.068]). Also, we reported a negative and statistically significant relationship between perceived severity of a health problem and healthcare utilization (β= -0.034, IRR = 0.966, 95% CI= [0.953–0.980]). The negative association between perceived barriers to care-seeking and healthcare utilization achieved statistical significance (β= -0.020, IRR = 0.981, 95% CI= [0.962–0.999]). Moreover, there was a positive and statistically significant association between cues to action and healthcare utilization (β = 0.058, IRR = 1.060, 95% CI= [1.027–1.095]). Lastly, we observed a positive and statistically significant association between self-efficacy and healthcare utilization (β = 0.032, IRR = 1.033, 95% CI= [1.003–1.063]).

In the full Model (Model 4) which consisted of all variables in Model 3 plus a health-related variable,  the study revealed a positive and statistically significant association between perceived susceptibility to a health problem and healthcare utilization (β = 0.054, IRR = 1.056, 95% CI= [1.041–1.071]). Also, we reported a negative and statistically significant relationship between perceived severity of a health problem and healthcare utilization (β= − 0.040, IRR = 0.961, 95% CI= [0.947-0.975]). There was a positive and statistically significant association between cues to action and healthcare utilization (β = 0.076, IRR = 1.079, 95% CI= [1.044–1.114]). Lastly, we observed a positive and statistically significant association between self-efficacy and healthcare utilization (β = 0.042, IRR = 1.043, 95% CI= [1.013–1.074]).

Apart from the main dimensions of the HBM, other demographic, socio-economic and health-related factors were associated with healthcare utilization. For example, the study revealed that participants aged between 18 and 24 years (β= − 0.305, IRR = 0.737, 95% CI= [0.620-0.877]), 25–34 years (β= − 0.321, IRR = 0.725, 95% CI= [0.628-0.837]), 35–44 years (β= − 0.231, IRR = 0.794, 95% CI= [0.693-0.910]), 45–54 years (β= -0.222, IRR = 0.801, 95% CI= [0.699-0.917]) and 55–64 years (β= − 0.181, IRR = 0.835, 95% CI= [0.720-0.968]) significantly had a lower log count of healthcare utilization compared to those who were aged 65 years or above. The results further showed that participants with junior high school education significantly had a higher log count of healthcare utilization compared to those who had completed their tertiary education (β = 0.124, IRR = 1.132, 95% CI=[1.015-0.1.262]). The analysis demonstrated that being unemployed was associated with a lower log count of healthcare utilization (β= − 0.088, IRR = 0.916, 95% CI= [0.850-0.986]). The results established that participants who were not enrolled in the health insurance scheme had a lower log count of healthcare utilization compared to those who were enrolled in the scheme (β= -0.174, IRR = 0.841, 95% CI= [0.774-0.913]). The analysis showed that participants who self-rated their health as very poor/poor (β = 0.553, IRR = 1.738, 95% CI= [1.396–2.163]), fair (β = 0.521, IRR = 1.683, 95% CI= [1.438–1.969]), good (β = 0.46, IRR = 1.584, 95% CI= [1.438–1.745]) and very good (β = 0.302, IRR = 1.353, 95% CI= [1.246–1.470]) had a higher log count of healthcare utilization compared to those who self-rated their health as excellent (see Table 6).

**Table 6:** Poisson regression models on predictors of healthcare utilization among informal caregivers of older adults

VARIABLES	Model 1		Model 2		Model 3		Full Model (Model 4)	
	IRR [ 95% CI]	B	IRR [ 95% CI]	B	IRR [ 95% CI]	B	IRR [ 95% CI]	B
**Health Beliefs variables**								
Perceived susceptibility to a health problem	1.069 [1.054–1.083]***	0.067	1.061[1.047–1.076]***	0.059	1. 053 [1.038–1.068]***	0.051	1.056 [1.041–1.071]***	0.054
Perceived severity of a health problem	0.963 [0.949-0.979]***	− 0.038	0.965[0.951–0.978]***	− 0.036	0.966 [0.953–0.980]***	− 0.034	0.961 [0.947-0.975]***	− 0.040
Perceived benefits of care-seeking	1.023 [1.002–1.045]*	0.023	1.019[0.998–1.040]	0.018	1.017 [0.996–1.038]	0.017	1.003[0.982-1.025]	0.003
Perceived barriers to care-seeking	0.980 [0.961-0.998]*	− 0.021	0.976[0.957–0.994]*	− 0.025	0.981 [0.962–0.999]*	− 0.020	0.986 [0.967-1.005]	− 0.014
Cues to action	1.066 [1.032-1.100]***	0.064	1.066[1.033–1.101]***	0.064	1.060 [1.027–1.095]***	0.058	1.079[1.044–1.114]***	0.076
Self-efficacy	1.035[1.006–1.066]*	0.035	1.033[1.004–1.064]*	0.033	1.033 [1.003–1.063]*	0.032	1.043[1.013–1.074]**	0.042
**Demographic variables**								
***Place of Residence of caregivers***								
Rural			1.007 [0.946–1.071]	0.007	1.001 [0.940–1.067]	0.001	1.030[0.967-1.098]	0.030
Urban (ref)			1.00		1.00		1.00	
***Age (years) of caregivers***								
18–24			0.626[0.530–0.740]***	− 0.468	0.630 [0.531–0.748]***	− 0.462	0.737[0.620-0.877]***	− 0.305
25–34			0.661[0.576–0.759]***	− 0.414	0.657 [0.570–0.757]***	− 0.421	0.725[0.628-0.837]***	− 0.321
35–44			0.750[0.657–0.855]***	− 0.288	0.730 [0.637–0.835]***	− 0.315	0.794[0.693-0.910]***	− 0.231
45–54			0.783[0.685–0.894]***	− 0.245	0.769 [0.671–0.881]***	− 0.263	0.801[0.699-0.917]***	− 0.222
55–64			0.822[0.710–0.953]**	− 0.196	0.817 [0.705–0.947]**	− 0.202	0.835[0.720-0.968]*	− 0.181
65 or above(ref)			1.00		1.00		1.00	
***Gender of caregivers***								
Male			0.935 [0.869–1.005]	− 0.067	0.967 [0.897–1.043]	− 0.033	0.942 [0.873-1.016]	− 0.060
Female (ref)			1.00		1.00		1.00	
***Marital status of caregivers***								
Never married			0.911[0.811–1.024]	− 0.093	0.923[0.821–1.038]	− 0.080	0.927[0.824-1.042]	− 0.076
Currently married			0.968[0.887–1.056]	− 0.033	0.958 [0.877–1.046]	− 0.043	0.981[0.898-1.072]	− 0.019
Separated/widowed/ divorced (ref)			1.00		1.00		1.00	
**Socio-economic variables**								
***Education level of caregivers***								
No formal education					1.132[1.013–1.264]*	0.124	1.075[0.962-1.201	0.072
Primary education					1.135[0.989–1.303]	0.127	1.070[0.931-1.228]	0.067
Junior high School					1.189[1.067–1.325]**	0.173	1.132[1.015–1.262]*	0.124
Senior high school					1.060[0.949–1.185]	0.059	1.034[0.926-1.156]	0.034
Tertiary Education (ref)					1.00		1.00	
***Employment Status of caregivers***								
Unemployed					0.929 [0.862-1.000]	− 0.074	0.916[0.850-0.986]*	− 0.088
Employed					1.00		1.00	
***Income (GH¢) of caregivers***								
Less than 1000					1.081[0.955–1.223]	0.078	1.071[0.946-1.212]	0.068
1000–1999					0.993 [0.863–1.142]	− 0.007	1.005[0.873-1.157]	0.005
2000 or above					1.00		1.00	
***Health insurance enrollment of caregivers***								
No					859[0.790–0.933]***	− 0.152	0.841[0.774-0.913]***	− 0.174
Yes					1.00		1.00	
**Health-related variable**								
***Self-rated health of caregivers***								
Very poor/poor							1.738[1.396–2.163]***	0.553
Fair							1.683[1.438–1.969]***	0.521
Good							1.584[1.438–1.745)***	0.460
Very good							1.353[1.246–1.470]***	0.302
Excellent (ref)							1.00	
**Model Fitness**								
Likelihood Ratio Chi-Square (p-value)	226.586 (0.000)		307.589 (0.000)		338.540 (0.000)		447.120 (0.000)	
Wald Chi-Square (p-value)	12.314 (< 0.001)		7.073 (0.008)		6.460 (0.011)		9.324 (0.002)	

*Test is significant at the 0.05 level

** Test is significant at the 0.01 level

*** Test is significant at the 0.001 level

## Discussion

This study was designed to investigate the association between the dimensions of the HBM and healthcare utilization among informal caregivers of older adults in the Ashanti Region of Ghana. Specifically, this study found statistically significant associations between healthcare utilization and four dimensions (perceived susceptibility to a health problem, cues to action, self-efficacy, and perceived severity of a health problem) of the HBM.

More importantly, the analysis showed a positive and significant association between perceived susceptibility to a health problem and healthcare utilization. Interestingly, our findings are comparable to a study by Luquis and Kensinger [64] which found an association between perceived susceptibility to a health problem and the utilization of preventive services among young adults in the United States. The key argument of the perceived susceptibility dimension of the HBM is that individuals who are more susceptible to a disease will be more likely to take action to address their health problems [53, 60, 61, 63, 64].

Our findings confirmed our first hypothesis that perceived susceptibility to a health problem is positively and significantly associated with healthcare utilization. We attribute our findings to the higher possibility of fear of sustaining health problems and the potential health complications for not seeking care. The takeaway message is that higher perceived susceptibility to a health problem increases healthcare utilization among informal caregivers of older adults.

Moreover, the analysis has demonstrated a positive and significant association between cues to action and healthcare utilization. Inconsistent conclusions have been reported on the association between cues to action and healthcare utilization in previous studies. For instance, Zhang et al. [86] did not establish any association between cues to action and periodic health examination in China. However, consistent with our results, Joiner et al. [87] reported an association between cues to action and enrollment in the National Diabetes Prevention Programme among insured adults with pre-diabetes in the United States. Our findings also agree with the key argument of the cues to action component of the HBM [53, 60, 68]. Further, a study from Ethiopia found that women with higher cues to action for institutional delivery of service use have higher odds of delivering at a health facility than those with lower cues to action [88]. A study by Schulz et al. [89] found that higher cues to action are associated with higher seeking of hearing evaluation services which is in line with our findings.

Our findings further confirmed our fifth hypothesis that the association between cues to action and healthcare utilization achieves positive and statistical significance. Our results indicate that a better understanding of the rationale of discovering health problems early, maintaining good health and participating in activities as indicators used to measure cues to action are integral to improving healthcare utilization among informal caregivers of older adults if health stakeholders pay attention to them.

This study found a positive and significant association between self-efficacy and healthcare utilization. This observation supports our sixth hypothesis that self-efficacy is positive and significantly associated with healthcare utilization. Consistent with our findings is a study by Moorthy et al. [84] which reported a positive association between self-efficacy and the use of self-protective measures to prevent the spread of COVID-19. Self-efficacy is conceptualized as an individual’s self-confidence to perform health-related behaviour [90]. Hence, having a higher self-efficacy enables individuals to have a higher likelihood of performing health-related behaviour [68, 91]. In this context, it is not surprising to report a direct association between self-efficacy and healthcare utilization since most of the study participants were able to determine when they have a health problem and tell when and where to seek healthcare. Health programmes intended to improve healthcare utilization among informal caregivers of older adults should therefore consider their self-efficacy.

We found a negative and significant association between perceived severity of a health problem and healthcare utilization. This finding was contrary to our second hypothesis of a positive relationship between perceived severity of a health problem and healthcare utilization. The negative association may be because at the time of the survey, many of our participants did not have any serious health problems which required urgent medical attention. In our descriptive analysis, most of the participants (94.7%) self-rated their health status as good/very good/excellent. Apart from the above, there may be other structural and systemic factors- high costs of care, non-enrollment in a health insurance scheme, transportation barriers, poor social/family support, and communication barriers- which impede healthcare utilization regardless of the severity of a health problem.

A study from China found a positive relationship between perceived severity of HIV/AIDs and the use of condoms [80]. The disparities in the findings may be due to differences in the units of analysis, the conceptualization of healthcare utilization and self-rated health. The HBM argues that there may be higher severity of a health problem; however, the presence of some barriers may deter health-related behaviour [60, 63, 91]. A previous study of why people avoid medical care has reported high cost of care, non enrollment in health insurance scheme and limited time as factors preventing people from seeking healthcare [92]. More importantly, our results offer opportunities for future studies to explore the underlying reasons for the negative association between perceived severity of a health problem and healthcare utilization among informal caregivers of older adults in Ghana and elsewhere.

Apart from the dimensions of the HBM, several demographic, socio-economic, and health-related characteristics influence healthcare utilization. For instance, our analysis showed that age, education level, employment status, health insurance enrollment and self-rated health of caregivers predicted healthcare utilization. This is consistent with previous studies that have established that age [47], education [37, 45], employment [93, 94], health insurance enrollment [51, 95] and self-rated health [76] are associated with healthcare utilization.

The strengths and limitations of this study also need comment. This study is the first to examine factors associated with healthcare utilization among informal caregivers of older adults in the Ashanti Region of Ghana using the constructs of the HBM. The study thus contributes greatly on both empirical and theoretical grounds regarding healthcare utilization among informal caregivers of older adults. Second, the study employed a large sample size (N = 1853) of informal caregivers from 13 districts made up of 39 communities (18 rural and 21 urban) in the Ashanti Region of Ghana.

Despite these strengths, there are some important limitations of the study which are highlighted. One, the study was cross-sectional and so we are unable to draw any causal relationships between the various dimensions of the HBM and healthcare utilization. Two, although we employed a large sample size of 1,853 informal caregivers, all reside in one region of Ghana which limits the generalization of our findings. Last, we employed a snowballing sampling technique to recruit our participants which has a higher likelihood of increasing the likelihood of minor biases in the data.

### Implications for policy, practice, and future research

In terms of policy, the implementation of the findings from this study may contribute partly to the realization of the United Nations’ health-related Sustainable Development Goals and targets in Ghana particularly in the study area. Also, the findings from this study may inform policy development towards the attainment of universal health coverage in Ghana specifically in the study area. Based on the findings of this study, the development of health policy and programmes to improve healthcare utilization among informal caregivers of older adults in the study area should extend beyond the significant dimensions of the HBM to include other significant demographic, socio-economic and health-related factors. In terms of practice, the findings from this study offer opportunities for healthcare professionals to understand factors associated with healthcare utilization among informal caregivers of older adults in the study region. Such knowledge is needed to promote healthcare delivery among informal caregivers of older adults in the study region. Also, organizing frequent health education programmes by health stakeholders at both the community level and healthcare facilities on health motivations (cues to action) and self-efficacy in the study region are welcomed since such measures may improve healthcare utilization among informal caregivers of older adults. Besides, improving informal caregivers’ awareness of their perceived susceptibility to health issues through health education and training programmes can shape healthcare utilization in the study area.

Since this study is quantitative, we could not account for other qualitative factors influencing healthcare utilization among informal caregivers of older adults in the study region. Against this background, we suggest that future studies investigate healthcare utilization among informal caregivers of older adults using a mixed methods design. Also, given that this study was not able to capture the causal effects of the association between the dimensions of the HBM, demographic, socio-economic and health-related factors in relation to healthcare utilization, future studies on healthcare utilization among informal caregivers of older adults may benefit from a longitudinal analysis.

## Conclusion

Findings from this study to a large extent support the association between the dimensions of the HBM and healthcare utilization among informal caregivers of older adults in the Ashanti Region of Ghana. Our findings, therefore, underline the importance of incorporating significant demographic, socio-economic and health-related factors along with the various dimensions of the HBM in any health policies and programmes intended to improve healthcare utilization among informal caregivers of older adults. Our study also emphasizes the need for more research on healthcare utilization among informal caregivers in national, regional, and local settings in developing countries given the challenges governments are facing as the population of older adults increases.

## Abbreviations

AIDS: Acquired Immunodeficiency Syndrome
CHRPE: Committee on Human Research Publication and Ethics
GREB: General Research Ethics Board
HBM: Health Belief Model
HIV: Human Immunodeficiency Virus
KNUST: Kwame Nkrumah University of Science and Technology
SE: Standard Error
SSA: Sub-Saharan Africa
SPSS: Statistical Package for the Social Sciences
VIF: Variance Inflation Factor

## Declarations

### Availability data and materials

The datasets used and/or analysed during the current study are available from the corresponding author on reasonable request.
